# Ringworm in calves: risk factors, improved molecular diagnosis, and therapeutic efficacy of an *Aloe vera* gel extract

**DOI:** 10.1186/s12917-020-02616-9

**Published:** 2020-11-04

**Authors:** Yasmine H. Tartor, Wafaa M. El-Neshwy, Abdallah M. A. Merwad, Mohamed F. Abo El-Maati, Rehab E. Mohamed, Hesham M. Dahshan, Hala I. Mahmoud

**Affiliations:** 1 Department of Microbiology, Faculty of Veterinary Medicine, Zagazig University, Zagazig, 44511 Egypt; 2 Infectious Diseases, Department of Animal Medicine, Faculty of Veterinary Medicine, Zagazig University, Zagazig, 44511 Egypt; 3 Department of Zoonoses, Faculty of Veterinary Medicine, Zagazig University, Zagazig, 44511 Egypt; 4 Department of Biochemistry, Faculty of Agriculture, Zagazig University, Zagazig, 44511 Egypt; 5 Department of Veterinary Public Health, Faculty of Veterinary Medicine, Zagazig University, Zagazig, 44511 Egypt; 6 Department of Animal Wealth, Faculty of Veterinary Medicine, Zagazig University, Zagazig, 44511 Egypt

**Keywords:** Calves dermatophytosis, Risk factors, Direct-sample nested PCR, Antifungal drugs, *Aloe vera* gel extract, Treatment

## Abstract

**Background:**

Dermatophytosis in calves is a major public and veterinary health concern worldwide because of its zoonotic potential and associated economic losses in cattle farms. However, this condition has lacked adequate attention; thus, to develop effective control measures, we determined ringworm prevalence, risk factors, and the direct-sample nested PCR diagnostic indices compared with the conventional methods of dermatophytes identification. Moreover, the phenolic composition of an *Aloe vera* gel extract (AGE) and its in vitro and in vivo antidermatophytic activity were evaluated and compared with those of antifungal drugs.

**Results:**

Of the 760 calves examined, 55.79% (424/760) showed ringworm lesions; 84.91% (360/424) were positive for fungal elements in direct-microscopy, and 79.72% (338/424) were positive in culture. *Trichophyton verrucosum* was the most frequently identified dermatophyte (90.24%). The risk of dermatophytosis was higher in 4–6-month-old vs. 1-month-old calves (60% vs. 41%), and in summer and winter compared with spring and autumn seasons (66 and 54% vs. 48%). Poor hygienic conditions, intensive breeding systems, animal raising for meat production, parasitic infestation, crossbreeding, and newly purchased animals were statistically significant risk factors for dermatophytosis. One-step PCR targeting the conserved regions of the 18S and 28S genes achieved unequivocal identification of *T. verrucosum* and *T. mentagrophytes* in hair samples. Nested-PCR exhibited an excellent performance in all tested diagnostic indices and increased the species-specific detection of dermatophytes by 20% compared with culture. Terbinafine and miconazole were the most active antifungal agents for dermatophytes. Gallic acid, caffeic acid, chlorogenic acid, cinnamic acid, aloe-Emodin, quercetin, and rutin were the major phenolic compounds of AGE, as assessed using high-performance liquid chromatography (HPLC). These compounds increased and synergized the antidermatophytic activity of AGE. The treated groups showed significantly lower clinical scores vs. the control group (*P* < 0.05). The calves were successfully treated with topical AGE (500 ppm), resulting in clinical and mycological cure within 14–28 days of the experiment; however, the recovery was achieved earlier in the topical miconazole 2% and AGE plus oral terbinafine groups.

**Conclusions:**

The nested PCR assay provided a rapid diagnostic tool for dermatophytosis and complemented the conventional methods for initiating targeted treatments for ringworm in calves. The recognized antidermatophytic potential of AGE is an advantageous addition to the therapeutic outcomes of commercial drugs.

## Background

Fungal infections associated with zoonotic transmission are an important public health problem worldwide [1]. Cattle dermatophytosis is a major public and veterinary health concern, not only because of its high zoonotic impact, but also because of economic losses in cattle farms attributed to hide damage, loss of weight, decimated meat and milk, contagiousness among animals, treatment costs, and difficulty to implement control measures [2, 3]. Ringworm is usually enzootic in cattle herds and is more prevalent in calves [2]. This may be explained by stressors such as rapid growth, weaning, or parasite burden (which weaken their immunity and skin health), as well as close confinement, dietary factors (deficiencies), and production systems [4]. Importantly, *Trichophyton verrucosum* is the predominant zoophilic dermatophyte causative species of dermatophytosis in cattle and can occasionally spread to humans through direct contact with cattle or infected fomites, causing highly inflammatory skin and hair dermatophytoses [4–6]. Therefore, the development of a precise laboratory test for the identification of dermatophyte species is pivotal for the prevention and effective control of dermatophytoses [2]. In this context, research articles that addressed the prevalence, risk factors, and treatment of calves’ ringworm in Egypt are scarce.

Furthermore, literature on the direct molecular diagnostic assays that are used for the detection and identification of dermatophytes in animal clinical samples is lacking [7, 8], and there is a need to surpass the time-consuming conventional methods based on microscopy and fungal cultures, which require weeks [8]. The nested polymerase chain reaction (PCR) technique is an effective practical diagnostic approach for dermatophytosis that has helped clinicians initiate rapid and targeted, as opposed to empirical, treatments of animal ringworm [7].

Dermatophytosis in animals remains difficult to eradicate because of antifungal resistance, the scarcity of accessible and authorized antifungal agents for use in veterinary practice, the restricted systemic treatment of livestock because of hepatotoxicity, and drug residues in products consumed by humans [2, 9]. Thus, the discovery of natural, less-toxic, and more-specific therapeutic alternatives is gaining ground. However, the antidermatophytic potential of natural products is plagued by a lack of in vivo studies affirming the antifungal activity of bioactive compounds discovered using in vitro studies [9]. *Aloe vera* is a plant of the Liliaceae family that has multiple applications, including antifungal, antibacterial, antioxidant, and antiseptic properties and use in cosmetics industries [10]. Nevertheless, the investigations of the in vitro and in vivo antidermatophytic potential of *Aloe vera* and the determination of its bioactive compounds remain modest.

Hence, this work was designed to investigate (i) the prevalence and risk factors of calves’ ringworm in Egypt, (ii) the diagnostic indices of direct nested PCR for the detection and identification of dermatophyte species on hair and scale samples compared with those of the conventional microscopic and culture methods, (iii) the biological activity and phenolic composition of an *Aloe Vera* gel extract (AGE), (iv) the antifungal activity of AGE in comparison to the antifungal drugs, and (v) the application of AGE for the treatment of calves’ ringworm.

## Results

### Prevalence of dermatophytosis among clinically examined calves

On clinical examination, 55.79% of calves (424/760) showed grayish-white, crusty, circular, and circumscribed discrete lesions (Additional Fig. 1A); moreover, alopecic, erythematous areas that remained after the removal of raised greasy crusts were observed occasionally (Additional Fig. 1B). The skin lesions were mostly found on the head and neck (46.69%) and all over the body (44.81%). Some cases (8.49%) also had lesions on the head, neck, and trunk. The degree of infection varied from moderate (55.18%) to severe (44.81%).

Direct microscopic observation of skin scrapings and hair samples revealed the presence of ectothrix arthroconidia in 84.91% (360/424) of samples, whereas, 79.72% (338/424) were positive for mycological culture. Fungal culture resulted in the identification of *T. verrucosum* (90. 24%) and *T. mentagrophytes* (9.76%) from the infected animals.

### Potential risk factors for calves’ ringworm

As revealed in Table 1, there was a highly significant (*P* < 0.001) association between ringworm infection and the investigated risk factors. A significant effect of age was showed on likelihood of ringworm infection as the risk of infection was 2.201 times higher in 4–6-month-old animals vs. younger calves with relative risk ratio 1.481. Meanwhile, crossbred animals were more likely (5.558 times higher) to be infected compared to purebred ones with relative risk ratio 1.724.

**Table 1:** Final logistic regression model of potential risk factors significantly (*P* < 0.05) associated with ringworm infection in calves

Risk factor	Calves		Prevalence	***β***^**a**^	S.E.(***β***)	OR^**b**^	Relative risk
	Infected	Non-infected	(%)			(95% CI)	(95% CI)
**Age**							
1 month (ref.)	66	97	40.49				
4–6 months	358	239	59.97	0.789	0.18	2.201 (1.546–3.133)	1.481 (1.215–1.804)
**Breed**							
Pure breed (ref.)	297	312	48.77				
Crossbreed	127	24	84.11	1.715	0.236	5.558 (3.494–8.843)	1.724 (1.549–1.919)
**Season**							
Summer (ref.)	190	100	65.52				
Winter	81	69	54	−0.48	0.205	0.617 (0.413–0.923)	0.824 (0.695–0.976)
Spring	83	90	47.98	−0.72	0.196	0.485 (0.331–0.713)	0.732 (0.614–0.873)
Autumn	70	77	47.62	−0.74	0.206	0.478 (0.319–0.716)	0.726 (0.601–0.878)
**Area per calf**							
Overcrowding (1.5 m/calf) (ref.)	194	120	61.78				
No overcrowding (6 m/calf)	230	216	51.57	−0.42	0.149	0.659 (0.491–0.884)	0.834 (0.736–0.945)
**Ventilation**							
Good (ref.)	295	315	48.36				
Bad	129	21	86	1.88	0.248	6.559 (4.027–10.683)	1.778 (1.602–1.973)
**Production system**							
Milk (ref.)	282	318	47				
Meat	142	18	88.75	2.185	0.263	8.896 (5.310–14.902)	1.888 (1.706–2.089)
**Breeding system**							
Semi-intensive (ref.)	305	297	50.66				
Intensive	119	39	75.32	1.089	0.202	2.971 (2.001–4.412)	1.486 (1.319–1.674)
**Origin of the animals**							
Born at the farm (ref.)	302	313	49.11				
Newly purchased	122	23	84.14	1.704	0.241	5.497 (3.426–8.820)	1.713 (1.539–1.907)
**Parasitic infestation**							
Absent (ref.)	312	278	52.88				
Present	112	58	65.88	0.542	0.181	1.720 (1.205–2.456)	1.245 (1.091–1.422)
**Use of disinfectant**							
Irregular (ref.)	125	27	82.24				
Regular	299	309	49.18	−1.57	0.227	0.209 (0.133–0.326)	0.598 (0.536–0.667)

^a^Regression coefficient

^b^Odds ratio

The highest risk of calves’ dermatophytosis was observed in summer and winter compared with spring and autumn seasons (65.5 and 54% vs. 48%). During winter, spring, and autumn seasons, the animals were less likely to be infected compared with summer season with risk ratios 0.824, 0.732 and 0.726, respectively. The risk of infection in intensive breeding system, newly purchased animals introduced to the farm, and conditions of parasitic infestation was higher (2.971, 5.497, and 1.720 times, respectively) compared with semi-intensive breeding system, the animals born at the farm, and the absence of parasitic infestation with relative risk 1.486, 1.713, and 1.245, respectively. The bad ventilation resulted in significant increase (6.559 times) in the likelihood of ringworm infection compared to the good ventilation with risk ratio 1.778. The area available per calf highly affected the infection potential. A narrower area (1.5 m^2^/calf) led to a greater spread of skin lesions among the animals; however, the likelihood of ringworm infection for no overcrowding (6 m^2^/calf) decreased 0.659 times and the risk ratio was 0.834. Ringworm lesions were 8.896 times more likely in animals reared for meat production compared with animals reared for milk production with risk ratio 1.88. Also, regular use of disinfectant decreased the likelihood of ringworm infection significantly (0.209 times) compared with irregular system with risk ratio 0.589.

**Fig. 1:**
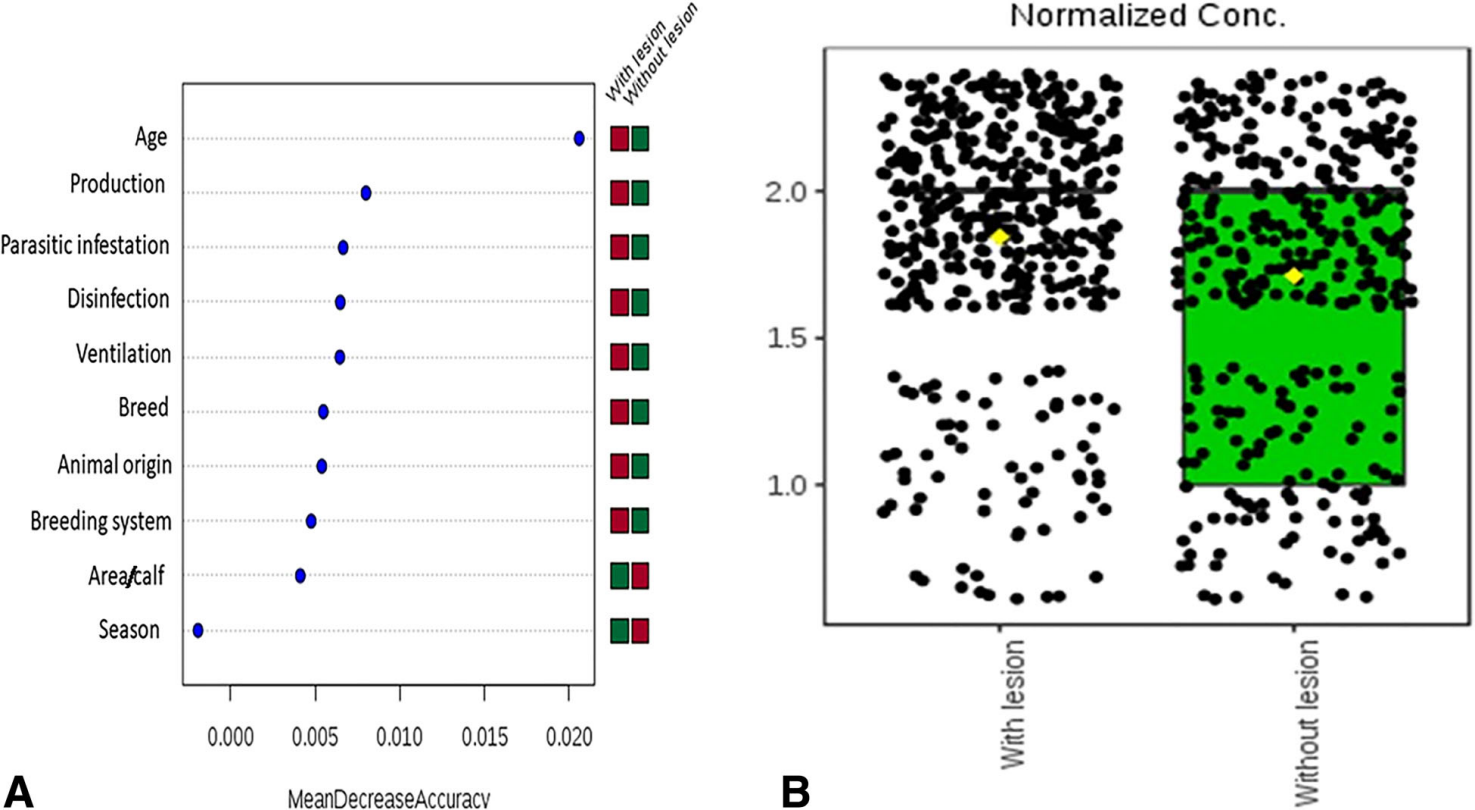
(**a**) Random forest classification showing the most important risk factor (y-axis) as a classifier differentiating between diseased and non-diseased calves when it was clinically examined. The X-axis refers to the predictive accuracy of the studied risk factors. The mini heatmap shows the frequency distribution of each factor across the two outcomes (ringworm lesion and without lesion). Each dot refers to the value of mean decrease accuracy of one risk factor, (**b**) Box plot for a normal distribution of age (as a continuous variable) across the examined calves (*n* = 760), each dot represents one case and the horizontal line refers to the median of age distribution

The random forest classification model and box plot (Fig. 1a and b) confirmed this observation, i.e. the age of calves was the most important risk factor, followed by the production system, presence of parasitic infestation, and irregular use of disinfectant was the fourth most risk factor.

### Nested PCR for the detection and identification of dermatophytes in clinical samples

**Fig. 2:**
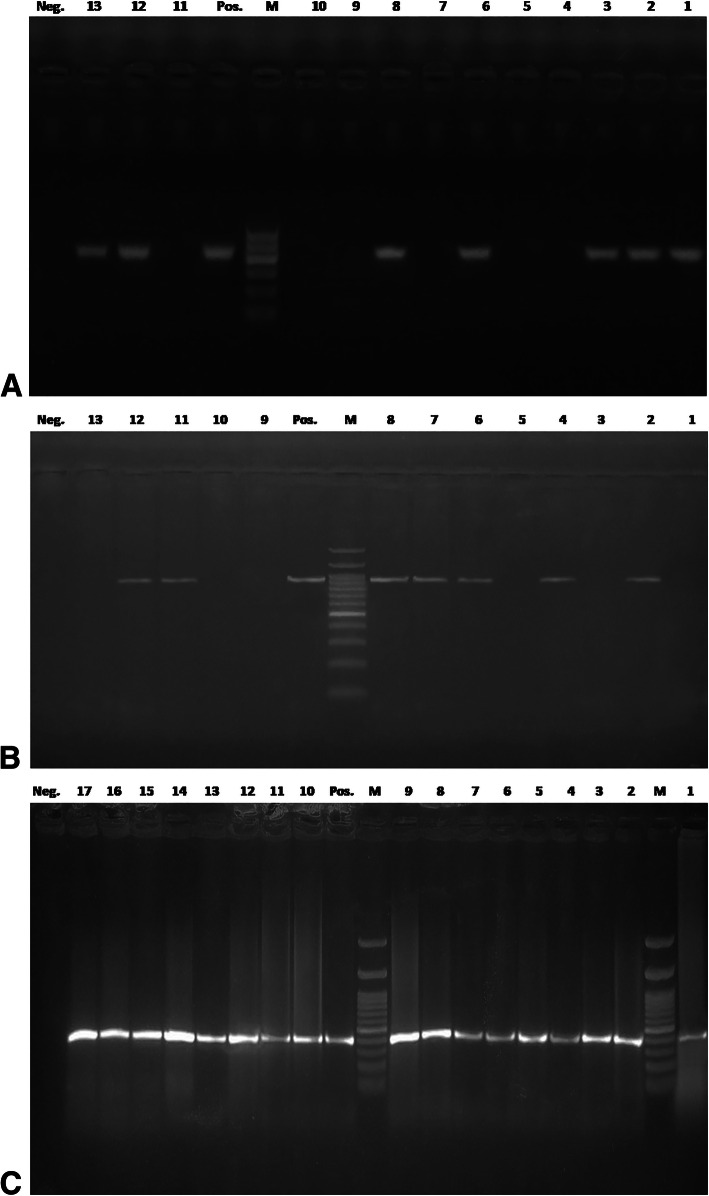
Agarose gel electrophoresis for amplicons of direct PCR assays for testing dermatophytes DNA from calves’ hair samples. (**a**) Pan-dermatophytes PCR amplicons of p*chs*-1gene at 440 bp, (**b**) One-step PCR ITS+ amplified products for *T. verrucosum* at 900 bp (lanes 2–8) and *T. mentagrophytes* at 872 bp (lanes 11, 12), and (**c**) ITS-1 amplicons of nested PCR at 400 bp. Lanes M: 100 bp molecular size marker, lane Pos.: positive control and lane Neg.: negative control

Pan-dermatophyte, one-step and nested PCR methods were evaluated in the context of dermatophyte identification in 75 samples that were direct microscopy and culture-positive, 36 samples that were positive by microscopy alone, nine samples positively diagnosed by culture alone, and 30 negative samples. The Pan-dermatophyte PCR could specifically detected dermatophyte DNA in 58%; one-step PCR did so in 62%; and nested PCR was positive in 72% of 150 samples (Table 2) with 440 bp p*chs*-1 amplicons, ∼ 900 bp ITS+ amplicons, and 400 bp ITS-1 amplicons, respectively (Fig. 2 a, b, and c).

**Table 2:** Results of direct microscopy, culture, and direct sample-PCR assays for the detection and identification of dermatophytes in 150 scales and hair samples

Direct microscopy	Dermatophyte culture	^a^Pan-dermatophyte PCR	^b^One-step PCR	Nested-PCR	Frequency (%)
[No. positive = 111 (74%)]	[No. positive = 84 (56%)]	[No. positive = 87 (58%)]	[No. positive = 93 (62%)]	[No. positive = 108 (72%)]	
**+**	**+**	**+**	**+**	**+**	51 (34)
**+**	**+**	**–**	**–**	**–**	6 (4)
**+**	**+**	**+**	**–**	**+**	9 (6)
**+**	**+**	**–**	**–**	**+**	9 (6)
**+**	**–**	**+**	**+**	**+**	6 (4)
**+**	**–**	**–**	**–**	**–**	15 (10)
**+**	**–**	**–**	**+**	**–**	6 (4)
**+**	**–**	**+**	**–**	**+**	3 (2)
**+**	**–**	**–**	**–**	**+**	6 (4)
**–**	**+**	**+**	**+**	**+**	9 (6)
**–**	**–**	**+**	**+**	**+**	6 (4)
**–**	**–**	**+**	**–**	**+**	3 (2)
**–**	**–**	**–**	**+**	**–**	15 (10)
**–**	**–**	**–**	**–**	**+**	6 (4)

Representative ^a^ p*chs-*1 and ^b^ ITS+ amplicons were sequenced for confirmation of the dermatophytes identification results. GenBank accession numbers of the nucleotide sequences were mentioned in the methods section

Nested PCR increased the species-specific detection of dermatophytes by 20 and 10% compared with culture alone or the combination of culture and direct microscopy, respectively.

Fungal culture identified dermatophytes in 56% (84/150), whereas direct microscopy identified dermatophytes in 74% (111/150) of samples. Out of the 66 samples that were negative for dermatophytes in culture, non- dermatophyte molds were cultured from 21 samples that were test-positive only by one-step PCR. In addition, non-dermatophyte molds were co-cultured with dermatophytes from six samples that were negative in the pan-dermatophyte, one-step, and nested PCRs.

As depicted in Table 3 the performance of the nested-PCR assay was excellent regarding all diagnostic indices tested. Using fungal culture as a reference standard, sensitivities of 82.14 and 71.43% and specificities of 72.73 and 50% were recorded for pan-dermatophyte and one-step PCR, respectively, whereas the corresponding values were 92.86 and 54.55% for nested PCR. In contrast, using the combination of culture and nested PCR as the gold standard, nested PCR was superior to the other methods as it achieved a sensitivity value of 94.74%, whereas culture and direct microscopy exhibited sensitivities of 73.68 and 78.95%, respectively. Specificity and PPVs were 100% for nested PCR and culture; therefore, they were considered as the gold standard, while the corresponding values were 41.67 and 81.08% for direct microscopy. Nested PCR was very accurate (AUC = 96%), whereas pan-dermatophyte PCR (82%) and culture (80%) were moderately accurate. A lower diagnostic accuracy was recorded for the direct microscopy and one-step PCR (50 < AUC ≤ 70%). The diagnostic odds ratio (DOR) of nested PCR is much higher than that of any other test which implies that the diagnostic performance of nested PCR was the best and was in strong agreement with the results obtained using culture and nested PCR results (Kappa value = 0. 91 and *P <* 0.001).

**Table 3:** Diagnostic indices of the direct-sample PCRs for the detection and identification of dermatophytes from ringworm lesions based on (a) culture and (b) culture and/or nested PCR as the gold standards

Diagnostic index^a^	Pan-dermatophyte	One step-PCR	Nested PCR	
**Culture as the gold standard**				
Se (95% CI)	82.14% (72.26–89.65)	71.43% (60.53–80.76)	92.86% (85.10–97.33)	
SP (95% CI)	72.73% (60.36–82.97	50% (37.43–62.57)	54.55% (41.81–66.86)	
PPV (95% CI)	79.31% (71.86–85.20)	64.52% (57.96–70.57)	72.22% (66.48–77.32)	
NPV (95% CI)	76.19% (66.40–83.82)	57.89% (47.58–67.56)	85.71% (72.91–93.05)	
LR+(95% CI	3.01 (2.01–4.52)	1.43 (1.08–1.88)	2.04 (1.56–2.68)	
LR- (95% CI)	0.25 (0.15–0.40)	0.57 (0.38–0.87)	0.13 (0.06–0.29)	
DOR (95% CI)	12.36 (5.64–26.7)	2.5 (1.72–4.19)	15.6 (5.965–40.799)	
AUC	78% (70.51–84.35)	62% (53.72–69.79)	76% (68.35–82.59)	
Kappa value	0.55***	0.217 **	0.493***	
	**Direct microscopy**	**Culture**	**One step-PCR**	**Nested PCR**
**Nested PCR and Culture as the gold standard**				
Se (95% CI)	78.95% (70.31–86.02)	73.68% (64.61–81.49)	63.16% (53.61–72.00)	94.74% (88.90–8.04)
SP (95% CI)	41.67% (25.51–59.24)	100% (90.26–100)	41.67% (25.51–59.24)	100% (90.26–100)
PPV (95% CI)	81.08% (76.19–85.16)	100%	77.42% (71.56–82.37)	100%
NPV (95% CI)	38.46% (26.99–51.38)	54.55% (46.88–62)	26.32% (18.47–36.02)	85.71% (73.36–92.89)
LR+(95% CI	1.35 (1.01–1.81)	^b^	1.08 (0.79–1.48)	^b^
LR- (95% CI)	0.51 (0.30–0.85)	0.26 (0.19–0.36)	0.88 (0.56–1.39)	0.05 (0.02–0.11)
DOR (95% CI)	2.68 (1.2–5.9)	0.46 (0.35–0.59)	1.22 (0.57–2.62)	19.6 (8.7–41.4)
AUC	70% (61.99–77.20)	80% (72.70–86.08)	58% (49.68–66)	96% (91.50–98.52)
Kappa value	0.2 **	0.57**	0.04	0.91***

^a^*Se* Sensitivity, *Sp* specificity, *PPV* positive predictive value, *NPV* negative predictive value, *LR+* positive likelihood ratio, *LR-* negative likelihood ratio, *DOR* diagnostic odds ratio and *AUC* accuracy

^b^Cannot be estimated/infinity

**, *** denote significant *P* values

Confirmatory DNA sequencing for the representative ITS+ and p*chs-*1 amplicons was performed, and the BLAST search of the resulting sequences produced hits that corresponded to *T. verrucosum* and *T. mentagrophytes* sequences available in the GenBank.

### Susceptibility of dermatophytes to antifungal drugs

The minimum inhibitory concentration (MIC) values of the five antifungal drugs for *T. verrucosum* and *T. mentagrophytes* are presented in Additional Table 1. The comparison of the values of the five antifungals for the two species tested revealed that those obtained for terbinafine were the lowest, followed by miconazole (MIC range, 0.03–0.25, 0.03–1 μg/mL; 0.06–0.5, 0.03–0.5 μg/mL, respectively). Moreover, the MIC_50_ and MIC_90_ values of terbinafine and miconazole were the lowest when compared with those of other antifungals. The mean MIC values ± SD of the tested antifungal agents did not differ between *T. verrucosum* and *T. mentagrophytes* (*P* > 0.05). Fluconazole was the least effective drug, with an overall MIC range of 8–64 μg/ mL.

### Yield, TPC, TF, phenolic compounds, antioxidant and antifungal activity of AGE

As depicted in Table 4 the AGE yield was 1.02 g extract of 100 g^− 1^. The amount of total phenolic compounds in AGE was 111.78 mg of gallic acid equivalent (GAE) g^− 1^ of gel. The flavonoid content of the extract was 45.6 mg of quercetin equivalent (QE) g^− 1^ of gel. Flavonoids have a broad range of chemical and biological activities, including radical-scavenging properties. For this reason, the extract was analyzed for total phenolic and flavonoid content. The major phenolic compounds of AGE were identified by HPLC and are presented in Table 4. They included gallic acid, caffeic acid, chlorogenic acid, cinnamic acid, aloe-Emodin, quercetin, and rutin. All compounds increased and synergized the antidermatophytic activity of AGE.

**Table 4:** Yield, TPC, TF and phenolic compounds of AGE

	Unit	^c^AGE
**Extract yield**	g/100 g *Aloe vera* gel	1.02 ± 0.052
^a^**TPC**	mg GAE/g extract	111.78 ± 10.62
^b^**TF**	mg QE/g extract	45.6 ± 8.45
**Phenolic compounds**		
Gallic acid	mg/ g AGE	0.12 ± 0.06
Caffeic acid		0.23 ± 0.11
Chlorogenic acid		0.54 ± 0.07
Cinnamic acid		0.98 ± 0.25
Aloe-Emodin		28.02 ± 4.67
Quercetin		1.54 ± 0.22
Rutin		1.14 ± 0.86

^a^*TPC* Total phenolic compounds, *GAE* gallic acid equivalent, ^b^ *TF* total flavonoids, *QE* quercetin equivalent and ^c^*AGE Aloe vera* gel extract

**Fig. 3:**
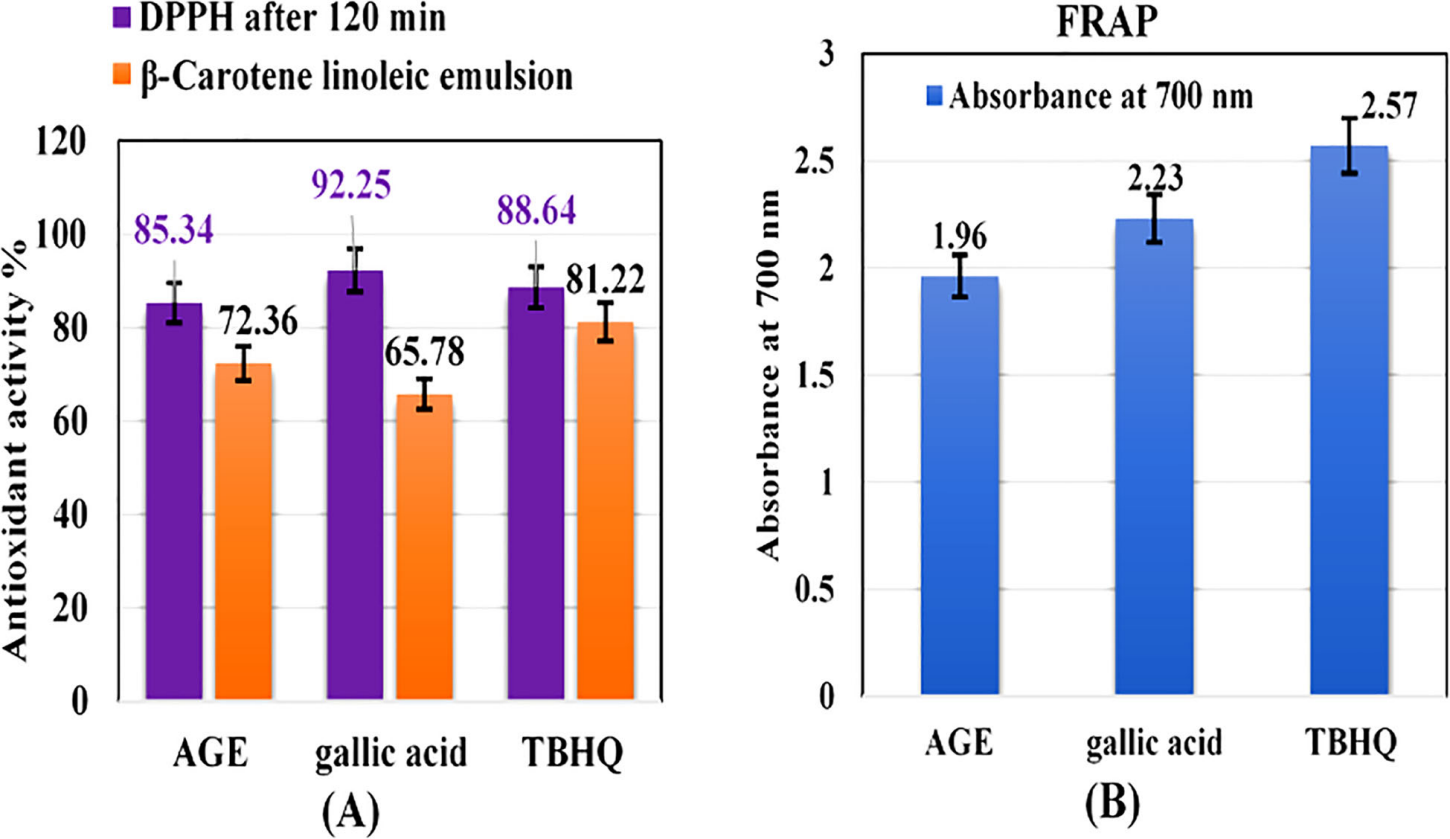
(**a**) The antioxidant activity of *Aloe vera* gel extract (AGE) against 1,1-Diphenyl-2picrylhydrazyl (DPPH˙) radical and β-Carotene/linoleic emulsion compared with gallic acid and tert-butyl hydroquinone (TBHQ), (**b**) The absorbance of ferric reducing power of AGE against gallic acid and TBHQ

The results of the analysis of the 1, 1-Diphenyl-2picrylhydrazyl (DPPH˙) radical-antioxidant activities of AGE are depicted in Fig. 3a and were as follows: antioxidant activity of 85.3% for AGE, 92.2% for gallic acid, and 88.6% for Tert-butyl hydroquinone (TBHQ) after 2 h of reaction. The results obtained demonstrated that this extract has antioxidant activity. As revealed in Fig. 3a, AGE inhibited the bleaching of *β*-carotene by scavenging linoleate-derived free radicals. The efficacy of the compounds was as follows (in decreasing order): TBHQ > AGE > gallic acid. The analysis showed a comparable scavenging ability between AGE (72.3%) and the synthetic antioxidants gallic acid (65.7%) and TBHQ (81.22%). AGE displayed a ferric reducing antioxidant power (FRAP) that was comparable to that of TBHQ and gallic acid (Fig. 3b). The FRAP of AGE was 1.96 vs. 2.23 for gallic acid and 2.57 for TBHQ. AGE exhibited an inhibitory effect for *T. verrucosum* and *T. mentagrophytes* at MIC values ranging from 300 to 400 ppm and 400 to 500 ppm, respectively.

### Effectiveness of AGE in the eradication of *T. verrucosum* from calves

In the treated calves, gradual improvement of the lesions was observed within 7–12 days post-treatment. Complete clinical recovery (full hair growth) was observed within 14–19 days after treatment for calves in G2 and G4 and within 21–28 days for animals in G1 and G3 (Additional Fig. 2). In contrast, the lesions detected on the control animals (G5) progressed and did not heal until 42 days of the study.

Direct microscopic examination and fungal cultures yielded negative results within the 4^th^ week of treatment. In contrast, samples from the untreated control calves repeatedly yielded positive mycological results during the investigation period.

**Fig. 4:**
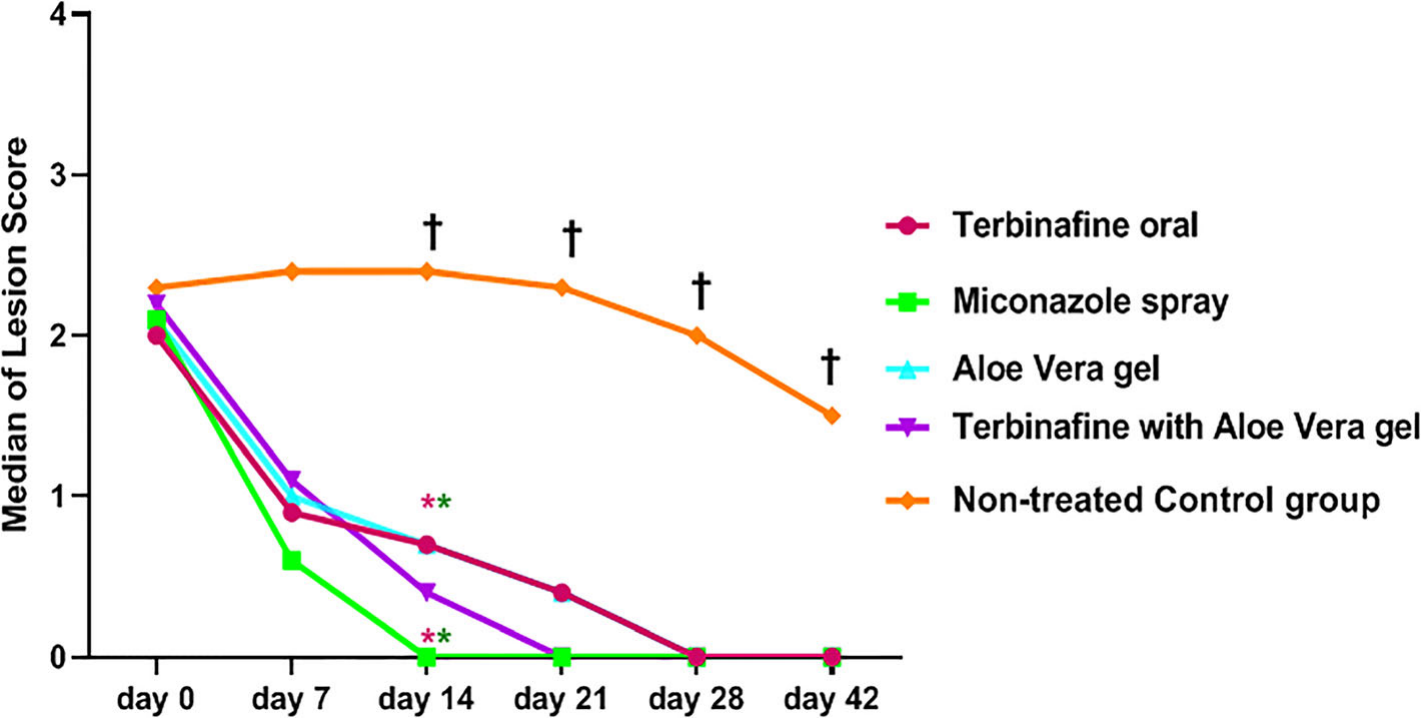
Medians for clinical scores of ringworm lesions on treated groups and control untreated group from day 0 to 42 days of the study. There is a non-significant difference between the clinical scores of groups on days 0 and 7, while the treated groups displayed significantly (*P* < 0.05) lower clinical scores than the control group on days 14, 21, 28, and 42. Clinical scores carrying asterisks with the same color was statistically different. **†** indicating a high significant difference between the control untreated group and all other groups within the same days

As revealed in Fig. 4, the clinical scores of the five animal groups did not differ significantly on days 0 and 7, while the treated groups showed significantly lower clinical scores than did the control (untreated) group (G5) on days 14, 21, 28, and 42 (*P* < 0.05).

On day 14, a significant improvement in the clinical scores (*P* < 0.05) was detected between miconazole treatment (G2) and the untreated control group (G5), G2 and terbinafine treatment (G1), and G2 and AGE (G3). Moreover, significant changes were detected between terbinafine treatment (G1) and the untreated group (G5) and AGE (G3) and G5 (Fig. 4).

Neither recurrence nor gross side effects were observed throughout the study period or during the clinical follow-up.

## Discussion

An enzootic circumstance of animal dermatophytosis is the outcome of the confinement of animals in breeding and the viability of the arthrospores in the environment for many months [2]. Prevention is difficult, but periodic surveys of the prevalence and risk factors of cattle ringworm may permit the adoption of increasingly effective prophylactic and control measures to prevent infection both to other animals and to humans [2, 11, 12]. In this study, the prevalence rate of ringworm in calves aged 1–6 months was 55.79%, that was nearly identical to that reported in Iran (57.5%) [5]. In contrast, the prevalence rate was higher than the 1.6% documented in Pakistan [12], but lower than the 87.7% reported in the Tuscany region [4] and the 71.7% observed in nearby Umbria, in Italy [11]. This discrepancy among countries is perhaps attributable to cattle breed, production, breeding system, origin of the cattle in the farm, and climatic conditions [11]. To answer the question which potential risk factor is most important and would best differentiate between the infected and non-infected clinically examined calves, we depended on the random forest classification model, which best suits doing this task (Fig. 1a) as demonstrated previously [13] *.* In accordance with other studies [4, 11], the random forest classification and box plot model indicated that age was the most important risk factor, as the risk of infection was higher in calves aged 4–6 month than it was in younger suckling calves (60% vs. 41%); and this could be attributed to the stressors of weaning and rapid growth. Furthermore, we found a highly significant correlation between several risk factors found in the examined calf population and ringworm infection, mainly season, bad ventilation, overcrowding, and irregular use of disinfectants. This reinforces the broadly accepted concept that high humidity, close contact between calves, poor hygienic conditions in stables play a significant role in the increase in ringworm prevalence [4, 5, 11, 12]. Hence, repeated topical treatment of all infected animals, together with good ventilation and thorough disinfection of stables, halters, fences, cleaning tools, and all of the materials that come into contact with the animals are the basis for the effective control of cattle ringworm [11, 14]. Of interest, there was a highly significant (*P* < 0.001) association between the risk of dermatophytosis and the new introduction of animals to the farm. In support of this finding, Papini et al. [4] debated that calves that are newly introduced into a herd spread the infection to both calves and humans, as they are carriers of dermatophytes before the development of clinical signs.

As described previously [2, 5, 11, 15], the detected clinical signs of cattle dermatophytosis were crusty lesions on the head and neck regions and other parts of the body. However, a study in Tanzania [16] reported occasion detection of the widespread lesions of alopecia and erythema which were also observed here. The detection rates were 84.91% by direct microscopy and 79.72% by fungal culture. In this context, inadequate scraping of the lesions and the slow and poor growth of *T. verrucosum* which hampered its detection, are probable explanations for the false-negative results of direct microscopy and culture, respectively [4]. According to previous studies [11, 15], *T. verrucosum* is the main dermatophyte causing cattle ringworm, although *T. mentagrophytes* which is usually associated with the presence of small rodents in farm has also been isolated in this context. The present findings showed that calves’ ringworm was caused by *T. verrucosum* in 90.24% and *T. mentagrophytes* in 9.76% of cases. Nevertheless, Aghamirian and Ghiasian [5] isolated *T. verrucosum* exclusively from 352 infected cows in Iran.

To date, molecular assays have been used for the detection of dermatophytes in clinical samples, as well as confirmation tests of the results of culture [17, 18]. Wollina and coauthors [17] have failed to cultivate *T. verrucosum* from the sample of a patient with severe tinea barbae. However, real-time PCR and ITS2 sequencing successfully detected *T. verrucosum*. No previous studies have attempted to identify dermatophyte species from hair samples of calves using a nested PCR assay. The findings obtained here revealed that one-step PCR correctly identified *T. verrucosum* and *T. mentagrophytes* in samples that were culture-positive (*n* = 72/150), with an amplicon size of 900 bp and 872 bp, respectively. In addition, nested PCR amplified ITS+ of both species in 108 samples and produced ITS-1 amplicons of 400 bp. This was in agreement with another study [7] that showed that the one step-PCR accurately identified *Microsporum canis* in hair samples from canines and felines with a band appearing at 922 bp, whereas ITS+ amplicons of 851–872 bp were obtained for *T. mentagrophytes*, *T. terrestre*, or *M. gypseum*; moreover, nested PCR achieved unequivocal identification of these species. The highly sensitive nested PCR method also exhibited a high specificity and PPV for detecting additional dermatophyte-positive samples that were missed by culture (*n* = 30/150) or by both microscopy and culture (*n* = 15). Other studies highlighted the incorporation of direct PCR in the laboratory diagnosis of onychomycosis for increasing the detection of dermatophyte-positive samples that are culture negative [19, 20]. A possible explanation for the low specificity and accuracy obtained using one-step PCR compared with pan-dermatophyte and nested PCR is the use of the universal fungal regions of rDNA. Moreover, the low sensitivity of the culture method could be attributed to the overgrowth of non-dermatophyte molds in the culture or the dermatophytes cultures were not yet positive after 4 weeks of incubation [4, 7]. Other reasons are the presence of non-viable fungal material in specimens from treated calves or that the DNA extraction step ease overcoming the impediment of trapping fungus in the keratin [20].

As reported previously [21, 22], terbinafine and miconazole were effective antifungal drugs for dermatophytes followed by itraconazole and griseofulvin; in contrast, fluconazole was the least-active antifungal agent. Recently, Pal [23] recommended the performance of further research for the development of cheap, safe, and potent chemotherapeutic agents for cattle dermatophytosis management. AGE is a cheap, easily obtainable, and safe natural product. Moreover, it is a limitless source of bioactive compounds with recognized antifungal activities that correlate with its antioxidant activities [24]. The results of the three assays used for measuring the antioxidant activity indicate that phenolic compounds have a high antioxidant capacity [25] because of their redox properties, which can play a significant role in the absorption and neutralization of free radicals, decomposition of peroxides, and quenching of singlet and reductive heavy metals with two or more valence states [26]. Phenolic compounds are the active antimicrobial constituents of various plants extract. However, the whole extract has a more noteworthy antifungal activity. Accordingly, AGE might be more advantageous than the isolated components, as the properties of a bioactive individual constituent can change its properties in the presence of other compounds [27]. The additive and synergistic effects of phenolic compounds account for their efficient bioactive properties, which explains why no single antimicrobial agent can supplant the combination of these natural components in achieving the antifungal activity [28]. The antidermatophytic activity of AGE recognized here was inconsistent with the findings of a previous report [29], which showed that the water extract of *Aloe vera* was effective against *T. mentagrophytes*. However, no reports exist of the activity of AGE against *T. verrucosum*. Nonetheless, most investigations were performed on fungal isolates, which hampers the extrapolation of the findings to real conditions. Therefore, additional in vivo studies are needed to ensure the reliability of the results [9]. The efficacy of the topical application of AGE for 2 weeks twice daily was compared with topical miconazole 2%, oral terbinafine with topical AGE and once-daily oral terbinafine in proven *T. verrucosum* infected calves. The clinical scores were significantly lower in all treated groups after 14 days of treatment compared with the untreated group (*P* < 0.05), whereas complete clinical recovery was achieved earlier in the miconazole group and AGE with oral terbinafine group vs. both the oral terbinafine group and AGE alone group. This indicates that the combination of AGE with oral terbinafine is effective for the treatment of dermatophytosis in calves. The results obtained were comparable with the findings of the treatment of calves with dermatophytosis with topical application of propolis and Whitfield’s ointment [30], as well as a polyherbal lotion combined with levamisole and griseofulvin [31].

## Conclusion

This study highlighted the need for good hygienic conditions, regular disinfection of holdings, rapid treatment of infected calves, and examination of the incoming calves to prevent dermatophytic epizoonoses in calves and humans. The implementation of a nested PCR assay provided a rapid diagnostic tool for dermatophytosis and complemented the conventional methods for dermatophyte-species-specific detection for the initiation of targeted treatment, thus reducing the burden of the economic losses caused by ringworm infection. The recognized antidermatophytic potential of AGE is an advantageous addition to commercial drugs and the combination of AGE with oral terbinafine has a potential therapeutic value against ringworm in calves.

## Methods

### Population and collection of clinical samples

From May 2015 to December 2018, a total of 760 Holstein cow calves (597 weaning and 163 suckling calves) raised in different farms in Egypt, were clinically examined for evidence of ringworm infection. Data about age, breed, farm production, breeding system, production management system, and the origin of calves of the farm were obtained for each calf as potential risk factors. For the assessment of parasitic infestation, fecal samples were examined for enteric parasites and thin blood films were prepared, fixed in absolute methyl alcohol, and stained with freshly filtered and diluted 10% Giemsa stain. After cleaning the skin lesion of the suspected ringworm-affected calf with 70% ethanol, scales and dull hair samples from the margins were collected using a sterilized plastic hair brush and tweezers, respectively [4].

Portions of hair and scales were examined microscopically after clearing with 20% potassium hydroxide (KOH), cultured on Mycobiotic Agar (Remel™, Thermo Fisher Scientific) slants with 10% thiamine and inositol, incubated at 30°C for 4–6 weeks, and observed for growth at 3-day intervals. Dermatophyte isolates were identified according to their macro-and micromorphological characteristics [32].

### Extraction of DNA from hair and scale samples and PCR amplification

The direct molecular identification of dermatophytes was executed in 150 clinical samples that were selected based on the results of direct microscopy and culture analyses. For the high-throughput disruption of samples, 50 mg of hair and scales were placed in a 2 mL safe-lock tube and incubated overnight at 55 °C with 360 μL of ATL buffer and 20 μL of QIAGEN protease (QIAamp DNA Mini kit, Qiagen, Germany, GmbH). Subsequently, tungsten carbide beads were added, and the tubes were placed into the TissueLyser adapter set for disruption using the TissueLyser for 2 min at 20–30 Hz twice. DNA extraction was performed using a QIAamp DNeasy Plant Mini kit (Qiagen, Germany, GmbH) according to the manufacturer’s instructions. DNA was eluted with 50 μL of elution buffer and the concentration was assessed using a NanoDrop™2000 spectrophotometer (Thermo Fisher Scientific, Waltham, MA, USA).

### One-step PCR

According to Cafarchia et al. [7] the chitin synthase (p*chs*-1) gene was amplified using DMTFchsF1 (5-CGAGTACATGTGCTCGCGCAC-3) and DMTFchsR1 (5-CGAGGTCAAARGCACGCCAGAG-3) primers to assess the presence of dermatophyte amplifiable DNA in the clinical samples. Subsequently, one-step PCR was performed using the primers DMTF18SF1 (5-CCAGGGAGGTTGGAAACGACCG-3) and DMTF28SR1 (5-CTACAAATTACAACTCGGACCC-3), which amplified a 900 bp fragment of the conserved regions in the 18S and 28S genes, which included the internal transcribed spacer regions of ribosomal DNA (ITS-1, 5.8S, and ITS-2).

### Nested PCR

A nested PCR was applied to amplify 400 bp of a conserved region in the dermatophyte 5.8S gene from the ITS+ amplicons of the primary PCR using the DMTF18SF1 and DMTFITS1R (5-CCGGAACCAAG AGATCCGTTGTTG-3) primers [7].

PCR was performed in an amplification reaction containing 12.5 μL of EmeraldAmp Max PCR Master Mix (Takara, Japan), 1 μL of each primer (20 pmol), 6 μL of DNA template in the case of primary PCR or 1 μL of diluted product from the primary PCR (dilution, 1:1 with molecular-grade water) for nested PCR and nuclease-free water was added up to 25 μL. *T. verrucosum* ATCC®28203™ and an amplification reaction without DNA template were used as a positive and negative control, respectively. Thermocycling conditions described previously [7] were used in an Applied Biosystems 2720 thermal cycler (Thermo Fisher Scientific, USA).

The amplified products were electrophoresed on ethidium-bromide-stained 1.5% agarose gels (Applichem, Germany, GmbH). A gelpilot 100 bp DNA ladder (Qiagen, Gmbh, Germany) and a 100 bp DNA ladder H3 RTU (Genedirex, Taiwan) were used to determine the amplicon sizes. A gel documentation system (Alpha Innotech, Biometra) was used to photograph the gels and the analysis of the data was performed using a computer software.

### DNA sequencing and sequence analysis

Thirty-seven representative ITS+ and p*chs*-1 PCR products were purified using the QIAquick PCR Product extraction kit (Qiagen, Valencia) and then sequenced using a Bigdye Terminator V3.1 cycle sequencing kit (Perkin-Elmer) in an Applied Biosystems 3130 genetic analyzer (HITACHI, Japan). DNA sequences were compared with those available in the National Center for Biotechnology Information (www.ncbi.nlm.nih.gov) database using the Basic Local Alignment Search Tool (BLAST). MEGA5 program, product version 5.1 (www.megasoftware.net) was used for sequence analysis. The ITS sequences were available under the GenBank accession numbers MK918485, MK918486, MT261760–MT261763, MT261765–MT261768, MT260175, MT260404, MT260449, MT260803, MT260878, MT261110, MT261113, MT261177, MT261197, MT261198, MT261202, MT261203, MT261385, MT261616, MT261746, MT261759, and MT269023. In addition, MT273253– MT273262 were for p*chs*-1 gene sequences.

### Antifungal susceptibility testing of dermatophytes isolates

The broth micro-dilution method (according to CLSI M38-A2 guidelines [33]) was used for testing the sensitivity of the dermatophyte isolates to the most commonly used antifungal drugs. Fluconazole was obtained from Pfizer International (New York, NY, USA), itraconazole, and miconazole were obtained from the Janssen Research Foundation (Beerse, Belgium), griseofulvin was purchased from Sigma Chemical Company (St. Louis, MO, USA), and terbinafine was purchased from Novartis (Basel, Switzerland). All drugs were dissolved in dimethyl sulfoxide (DMSO, Sigma-Aldrich), with the exception of fluconazole, which was dissolved in RPMI1640 medium (Sigma Co. St. Louis, USA), buffered at pH 7.0 with 165 mM of 3-(N-morpholino) propanesulfonic acid (MOPS; Sigma), and serially diluted two-fold to final concentrations of 0.125–64 μg/mL for fluconazole and 0.03–16 μg/mL for the other antifungal agents. MIC values, MIC_50_, and MIC_90_ were determined.

### Preparation of *Aloe vera* gel extracts (AGEs)

*Aloe vera* leaves were obtained from the Agriculture Faculty, Zagazig University, Zagazig, Egypt. *Aloe vera* gel was obtained from the leaves by scratching. The aqueous extract of the gel was prepared using a magnetic stirrer (Fisher Scientific) and filtered using Whatman No. 1 filter paper. The extraction ratio was 1:5 (W:V, gel:solvent). The filtrate was freeze-dried (Thermo-Electron Corporation-Heto power dry LL300 Freeze Dryer) and the extract was then weighed to decide the yield and stored at − 20 °C.

### Chemical characterization of AGE

#### Determination of phenolic compounds

The concentration of total phenols in the extract was measured by a UV spectrophotometer (Jenway-UV–VIS Spectrophotometer 6705) based on the colorimetric reduction of the reagent by phenolic compounds, as described by Škerget et al. [34]. The total phenolic content, expressed as GAE, was calculated as follows: **y = 0.0228 x + 0.0086 and** ***R***^**2**^ **= 0.9969**, where x is the concentration (μg GAE) and y is the absorbance.

#### Determination of total flavonoids

Total flavonoid content, expressed as QE, in AGE at a final concentration of 1 mg mL^− 1^ was calculated as follows: **y = 0.0142 x – 0.007** and ***R***^**2**^ **= 0.9994**, where x is the concentration (μg QE) and y is the absorbance [35].

### Determination of phenolic compounds by HPLC

The HPLC analysis was performed as described previously [36], with slight modifications, using an Agilent Technologies 1100 series liquid chromatograph equipped with an autosampler. The analytical column was Agilent Eclipse XDB C18 (100 × 4.6 μm; 3.5 μm particle size). The diode array detector was set to a scanning range of 180–420 nm. The mobile phase consisted of methanol (solvent A) and 0.1% formic acid (v/v) (solvent B). The flow rate was kept at 0.4 mL min^− 1^ and the gradient program was as follows: 10% A - 90% B (0–5 min); 20% A - 80% B (5–10 min); 30% A - 70% B (10–15 min); 50% A - 50% B (15–20 min); 70% A - 30% B (20–25 min); 90% A -10% B (25–30 min); 50% A -50% B (30–35 min); and 10% A - 90% B (35–36 min). A 5 min post-run was used for reconditioning. The injection volume was 10 μL and peaks were monitored simultaneously at 280, 320 and 360 nm for the benzoic acid and cinnamic acid (Sigma, St. Louis, MO, USA) derivatives and flavonoid compounds, respectively. All samples were filtered through a 0.45 μm Acrodisc syringe filter (Gelman Laboratory, MI) before injection. Peaks were identified based on congruent retention times and UV spectra and compared with those of the standards (Sigma, St. Louis, MO, USA).

### Antioxidant and biological activity of AGE

#### DPPH˙ radical-scavenging activity

The electron-donation ability of AGE was measured by bleaching of the DPPH˙ (Sigma, St. Louis, MO, USA) purple-colored solution using a UV spectrophotometer (Jenway-UV–VIS Spectrophotometer 6705) [37]. The absorbance was determined against the control at 515 nm [38]. The percentage of the scavenging activity of the DPPH˙ free radical was calculated as follows:

$$ \mathbf{Scavenging}\ \mathbf{activity}\ \left(\mathbf{Inhibition}\right)\%=\left[\left({\mathbf{A}}_{\mathbf{control}}-{\mathbf{A}}_{\mathbf{sample}}\right)/{\mathbf{A}}_{\mathbf{control}}\right]\times \mathbf{100}\operatorname{} $$

where A _control_ is the absorbance of the control reaction and A _sample_ is the absorbance in the presence of the plant extract. Gallic acid and TBHQ (Sigma, St. Louis, MO, USA) (1 mg/1 mL of methanol) were used as positive controls. Samples were tested in triplicate.

#### β-Carotene/linoleic acid bleaching

The ability of AGE and synthetic antioxidants (gallic aid and TBHQ) to hinder the bleaching of β-carotene (Sigma, St. Louis, MO, USA) was examined according to Dastmalchi et al. [39]. A control sample with no added extract was also analyzed. Antioxidant activity was calculated as follows:

$$ \mathbf{Antioxidant}\ \mathbf{activity}\ \left(\%\right)=\left[\mathbf{1}-\left({\mathbf{A}}_{\mathbf{sample}}^{\mathbf{0}}-{\mathbf{A}}_{\mathbf{sample}}^{\mathbf{120}}\right)/\left({\mathbf{A}}_{\mathbf{control}}^{\mathbf{0}}-{\mathbf{A}}_{\mathbf{control}}^{\mathbf{120}}\right)\right]\times \mathbf{100} $$

where **A**^**0**^_**sample**_ is the absorbance of the AGE or synthetic antioxidant at time 0, **A**^**120**^_**sample**_ is the absorbance after 120 min, and **A**^**0**^_**control**_ and **A**^**120**^_**control**_ are the absorbances of the control at time 0 and after 120 min, respectively.

#### Ferric reducing antioxidant power

The reducing power of the extract was assessed [38]. Distilled water was used as a negative control and gallic acid and TBHQ were used as positive controls. The absorbance of this mixture was measured at 700 nm using a UV spectrophotometer (Jenway-UV–VIS Spectrophotometer 6705). A decrease in absorbance indicated the ferric reducing power capability of the sample.

### Testing the antidermatophyte activity of AGE

The procedure of Silva et al. [40] was used to test the antidermatophyte activity of AGE. The freeze-dried AGE (3.5 g) was dissolved and serially two-fold diluted in RPMI-1640 broth, to obtain a concentration range of 1000–20,000 μg/mL as TPC. A final concentration of 50–1000 μg/mL was obtained by mixing 2 mL of this solution with 18 mL of liquefied Mycobiotic Agar medium (Remel™, Thermo Fisher Scientific) at 45 °C in a sterile Petri dish. Subsequently, wells with a diameter of 3 mm were made in the center of this agar plate and filled with 10 μL of the fungal spore suspension (10^6^ CFU/mL) that was prepared from freshly cultured isolates. The plates were incubated for 5 days at 25 °C. The assay was carried out in triplicate and growth and drug controls were incorporated into the test. The concentration that inhibited the fungal growth was considered as the MIC.

### Investigation of AGE effectiveness for the treatment of calf ringworm

Seventy-five calves showing evident clinical signs of ringworm were used for the investigation of AGE effectiveness in comparison with antifungal drugs for the treatment of this condition after obtaining informed consent from the farm owners. The enrolled calves were positive on mycological examination and *T. verrucosum* was isolated from clinical samples. Sample size calculation at a 0.05 significance level and 80% power revealed that 15 calves per group (G) would have been required. Calves exhibiting an equivalent severity of lesions distributed on the head, neck, and body were allocated randomly into five groups by using random number generator. Animals in G1 were treated orally with 250 mg/day of terbinafine (Lamisil®; Novartis, Basel, Switzerland). The crust on the skin lesions was removed with a brush and topical miconazole (Janssen Research Foundation, Beerse, Belgium) (G2), AGE solution (500 ppm) (G3), or oral terbinafine in combination with AGE (G4) were applied twice a day for 2 weeks. Animals in G5 were left untreated (as controls). Calves were observed daily for 6 weeks. In the beginning, during and after the treatment, the clinical efficacy was assessed by scoring dermatophytosis lesions on a 0–3 scale using previously published criteria [41–43] (Additional Table 2). The scoring was performed by the same investigator who was blinded to the treatment groups. The scores for each evaluated area (e.g. head, neck, and body) were averaged as follows: Sum of scores assigned to all lesions on the area/ Number of lesions on this area. Average total score of each animal = Sum of scores assigned to all evaluated areas/ Number of affected areas [44]. The lesions were assessed on every examination. The mycological examination was performed every week until two consecutive fungal cultures gave negative results [30, 44]. The control animals were treated after the observation period.

Each animal group was housed in a separate well ventilated, open sided pen with sheltered area. All the pens received similar management conditions. Area per calf in each pen was not less than 4m^2^ to avoid the overcrowding. The pens were bedded with straw that was changed every 1–2 weeks and antifungal disinfection for the entire pen and all materials with which animals come in contact was performed using 0.2% enilconazole (Clinafarm® EC; Merck Animal Health USA).

### Data analysis

Various risk factors that were recorded on the whole set of the 760 clinically examined calves for ringworm lesions were included as independent variables in a multiple step-wise logistic regression model (PROC LOGISTIC, SAS Institute Inc. [45]). An approximate measure of relative risk was determined using odds ratio (the antilogarithm of the coefficient) with 95% confidence intervals. To confirm results and to identify the most important risk factor as a classifier that differentiated between infected and non-infected calves, a random forest non-parametric classification analysis was performed using the MetaboAnalystR web server [46]. Briefly, the occurrence of each variable was first used to build up a random forest classification model (an ensemble of 500 tree trials; out-of-bag (OOB) error = 0.6) in the respective outcome. The importance of the risk factor was determined by measuring the increase in the OOB error when the respective factor was permuted. The sensitivity, specificity, negative and positive predictive values, positive and negative likelihood ratio, and diagnostic odds ratio, which express the strength of the association between the test results and disease, with 95% confidence intervals for direct-sample PCR assays were estimated. All diagnostic indices were predestined based on (a) culture and (b) culture and nested PCR as the gold standard for the detection/identification of dermatophytes causing calves’ ringworm. The Kappa value was used to test the agreement between test results. Student’s t-test was used to compare the mean MIC values ± SD of each antifungal drug for the tested species. Kruskal–Wallis test was used to analyze the differences in clinical score changes among the treated and untreated groups over time after confirming the significance of Shapiro-Wilk test results [47]. The differences in clinical scores between groups were assessed by the Mann–Whitney U test after a significant result on the Kruskal–Wallis test. Significance was set at *P* < 0.05.

## Supplementary Information

**Additional file 1: Table S1.** Mean MICs ± SD, MIC range, MIC_50_, and MIC_90_ values of antifungal drugs for *T. verrucosum* and *T. mentagrophytes* isolates.

**Additional file 2: Table S2.** Scoring of ringworm lesions in calves according to Moriello et al. 2004; Lund and DeBoer, 2008; and Balikci et al. 2016.

**Additional file 3: Fig. S1.** Ringworm in calves due to *T. verrucosum* with typical grey-white raised crusty lesions on head and neck regions (A) and extensive alopecia, erythema, and scales that remained after removal of thick greasy crusts firmly attached to erect and matted hair over the back (B).

**Additional file 4: Fig. S2.** Treatment of ringworm in calf with 500 ppm topical AGE (A) calf at day 0 before treatment and (B) after 20 days post-treatment.

## Data Availability

The datasets generated and/or analysed during the current study are available in the GenBank under accession numbers: MK918485, MK918486, MT261760–MT261763, MT261765–MT261768, MT260175, MT260404, MT260449, MT260803, MT273253–MT273262, MT260878, MT261110, MT261113, MT261177, MT261197, MT261198, MT261202, MT261203, MT261385, MT261616, MT261746, MT261759, and MT269023.

## Abbreviations

ITS: Internal transcribed spacers regions of ribosomal DNA
p*chs*-1: Partial chitin synthase
MIC: Minimum Inhibitory Concentration
AGE: *Aloe vera* gel extract
TPC: Total phenolic compounds
TF: Total flavonoids
QE: Quercetin equivalent
GAE: Gallic acid equivalent
PPV: Positive predictive value
NPV: Negative predictive value
AUC: Accuracy
DOR: Diagnostic odds ratio
LR: Likelihood ratio
