# Supplementary material for: Ringworm in calves: risk factors, improved molecular diagnosis, and therapeutic efficacy of an Aloe vera gel extract
Source: BMC Vet Res. 2020 Nov 4;16:421. doi: 10.1186/s12917-020-02616-9 (PMC7640396; doi:10.1186/s12917-020-02616-9)
Supplement: Supplementary file 1 — Additional file 1: Table S1. Mean MICs ± SD, MIC range, MIC50, and MIC90 values of antifungal drugs for T. verrucosum and T. mentagrophytes isolates. [file 12917_2020_2616_MOESM1_ESM.doc]

Additional Table (1): Mean MICs ± SD, MIC range, MIC_50_ and MIC_90_ values of antifungal drugs for *T. verrucosum and T. mentagrophytes* isolates.

| Antifungal agents | *T. verrucosum* | | | | *T. mentagrophytes* | | | | |
| --- | --- | --- | --- | --- | --- | --- | --- | --- | --- |
|  | Mean MIC ± SD  (µg ⁄ mL) | MIC range (µg ⁄ mL) | MIC_50_  (mg ⁄ mL) | MIC_90_  (µg ⁄ mL) | Mean MIC ± SD  (µg ⁄ mL) | MIC range  (µg ⁄ mL) | MIC_50_  (µg ⁄ mL) | MIC_90_  (µg ⁄ mL) | *P*-value |
| Terbinafine | 0.116 ± 0.097 | 0.03 - 0.25 | 0.06 | 0.25 | 0.234 ± 0.194 | 0.06-0.5 | 0.25 | 0.5 | 0.160 |
| Griseofulvin | 1.875 ± 1.547 | 0.5 - 4 | 2 | 4 | 0.937± 0.773 | 0.25-2 | 1 | 2 | 0.160 |
| Itraconazole | 2.333 ± 1.527 | 1 - 4 | 2 | 4 | 0.583 ± 0.381 | 0.25-1 | 0.5 | 1 | 0.063 |
| Miconazole | 0.327 ± 0.371 | 0.03 - 1 | 0.125 | 0.5 | 0.193 ± 0.191 | 0.03-0.5 | 0.125 | 0.5 | 0.242 |
| Fluconazole | 0.373 ± 24.440 | 16 - 64 | 0.16 | 64 | 18.66 ± 12.220 | 8-32 | 16 | 32 | 0.151 |
