# Supplementary material for: Ringworm in calves: risk factors, improved molecular diagnosis, and therapeutic efficacy of an Aloe vera gel extract
Source: BMC Vet Res. 2020 Nov 4;16:421. doi: 10.1186/s12917-020-02616-9 (PMC7640396; doi:10.1186/s12917-020-02616-9)
Supplement: Supplementary file 2 — Additional file 2: Table S2. Scoring of ringworm lesions in calves according to Moriello et al. 2004; Lund and DeBoer, 2008; and Balikci et al. 2016. [file 12917_2020_2616_MOESM2_ESM.doc]

**Additional Table 2: Scoring of ringworm lesions in calves according to Moriello et al. 2004; Lund and DeBoer, 2008; and Balikci et al. 2016**

| **Score** | **Characteristic skin lesion** | | | |
| --- | --- | --- | --- | --- |
|  | **Alopecia** | **Scales/Crust** | **Form** | **Erythema** |
| 0 | None | None | None | None |
| 1 | 1 mm in diameter | Fine scaling noticeable only on close examination | Circumscribed | Faint red; noticeable only on close examination |
| 2 | 2-3 mm | Easily visible scaling, minimal crust | Circumscribed | Bright red; easily visible upon close examination |
| 3 | ˃ 3 mm | Crusting with Keratinization | Diffused | Sever erythema; easily visible from a 1 meter distance |
