# Supplementary figures and images for: Ringworm in calves: risk factors, improved molecular diagnosis, and therapeutic efficacy of an Aloe vera gel extract
Source: BMC Vet Res. 2020 Nov 4;16:421. doi: 10.1186/s12917-020-02616-9 (PMC7640396; doi:10.1186/s12917-020-02616-9)

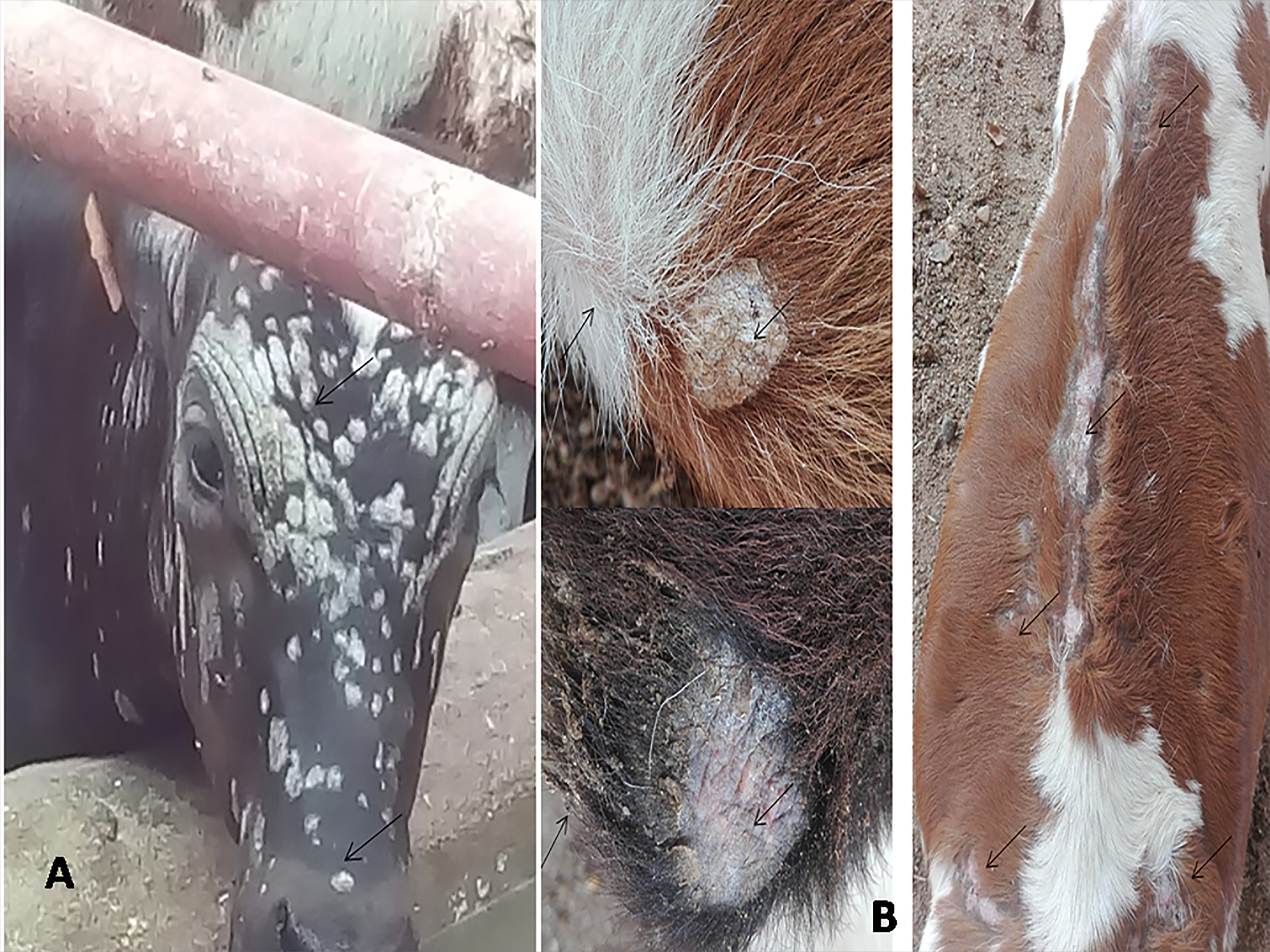

Supplement: Supplementary file 3 — Additional file 3: Fig. S1. Ringworm in calves due to T. verrucosum with typical grey-white raised crusty lesions on head and neck regions (A) and extensive alopecia, erythema, and scales that remained after removal of thick greasy crusts firmly attached to erect and matted hair over the back (B). [file 12917_2020_2616_MOESM3_ESM.tif]

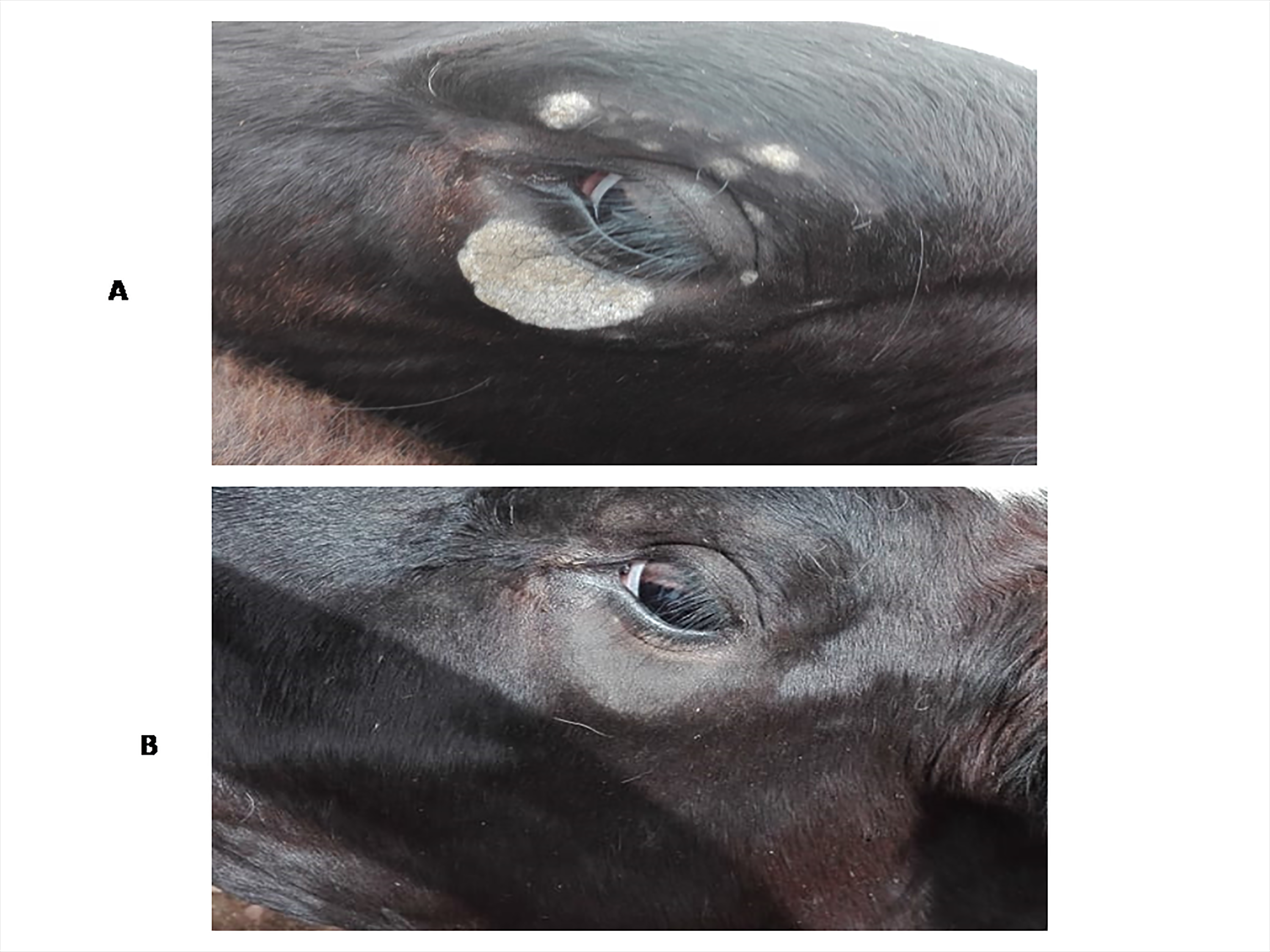

Supplement: Supplementary file 4 — Additional file 4: Fig. S2. Treatment of ringworm in calf with 500 ppm topical AGE (A) calf at day 0 before treatment and (B) after 20 days post-treatment. [file 12917_2020_2616_MOESM4_ESM.tif]
